# Meta-analysis identified the *TNFA *-308G > A promoter polymorphism as a risk factor for disease severity in patients with rheumatoid arthritis

**DOI:** 10.1186/ar4110

**Published:** 2012-12-07

**Authors:** Erik JM Toonen, Pilar Barrera, Jaap Fransen, Arjan PM de Brouwer, Agnes M Eijsbouts, Pierre Miossec, Hubert Marotte, Hans Scheffer, Piet LCM van Riel, Barbara Franke, Marieke JH Coenen

**Affiliations:** 1 Department of Medicine, Radboud University Nijmegen Medical Centre, Geert Grooteplein-zuid 10, Nijmegen, 6525 GA, The Netherlands; 2 Department of Rheumatology, Radboud University Nijmegen Medical Centre, Geert Grooteplein-zuid 10, Nijmegen, 6525 GA, The Netherlands; 3 Department of Human Genetics, Radboud University Nijmegen Medical Centre, Geert Grooteplein-zuid 10, Nijmegen 6525 GA, The Netherlands; 4 Department of Rheumatology, St Maartenskliniek, Hengstdal 3, Ubbergen, 6574 NA, The Netherlands; 5 Clinical Immunology Unit, Departments of Immunology and Rheumatology, hospital Edouard Herriot, Place 5 d'Arsonval, 69437, Lyon, France

## Abstract

**Introduction:**

The goal of this study is to investigate whether the -308G > A promoter polymorphism in the tumor necrosis factor alpha (*TNFA*) gene is associated with disease severity and radiologic joint damage in a large cohort of patients with rheumatoid arthritis (RA).

**Methods:**

A long-term observational early RA inception cohort (*n *= 208) with detailed information about disease activity and radiologic damage after 3, 6 and 9 years of disease was genotyped for the *TNFA *-308G > A promoter polymorphism (rs1800629). A longitudinal regression analysis was performed to assess the effect of genotype on RA disease severity and joint damage. Subsequently, a meta-analysis, including all publically available data, was performed to further test the association between joint erosions and the *TNFA *polymorphism. To learn more about the mechanism behind the effect of the polymorphism, RNA isolated from peripheral blood from RA patients (*n *= 66) was used for *TNFA *gene expression analysis by quantitative PCR.

**Results:**

Longitudinal regression analysis with correction for gender and disease activity showed a significant difference in total joint damage between GG and GA+AA genotype groups (*P *= 0.002), which was stable over time. The meta-analysis, which included 2,053 patients, confirmed an association of the genetic variant with the development of erosions (odds ratio 0.78, 95% CI 0.62, 0.98). No significant differences in *TNFA *gene expression were observed for the different genotypes, confirming earlier findings in healthy individuals.

**Conclusions:**

Our data confirm that the *TNFA *-308G > A promoter polymorphism is associated with joint damage in patients with RA. This is not mediated by differences in *TNFA *gene expression between genotypes.

## Introduction

TNF plays a key role in the pathogenesis of rheumatoid arthritis (RA) and other auto-immune diseases. Several genetic variants in the *TNFA *gene, which codes for TNFα, have been investigated in relation to disease severity, but none of them appears to be highly specific and/or sensitive. The most thoroughly investigated *TNFA *polymorphism is the -308G > A (rs1800629) located in the promoter of the gene, though studies focusing on this polymorphism have yielded conflicting results, [1-14].

Three studies [3,6,7] showed an association of the G allele with more pronounced radiologic joint damage. Other studies reported a worse radiologic outcome in patients carrying the A allele [1,9,13], or no association between the genetic variant and radiologic progression [2,8-12,14]. Given the contradictory results in these relatively small, individual studies, there is a need for a more comprehensive approach to evaluate the presence or absence of an association between the *TNFA *-308G > A polymorphism and the severity of RA and radiological joint damage.

As the -308G > A variant is located within the *TNFA *promoter, several studies have also investigated the polymorphism in relation to *TNFA *transcription, which could influence TNFα production and in turn, disease activity and severity [2,15-23]. An important study by Knight *et al*., using the haploChIP method for high-throughput screening of common DNA polymorphisms that (might) effect gene regulation *in vivo*, reported that the -308G > A polymorphism was not associated with *TNFA *expression in human B-cells [24]. A recent meta-analysis in healthy individuals (*n *= 1,825) shows that TNFα mRNA and protein levels are not influenced by the polymorphism [25].

Here, we first investigated the possible associations of the -308G > A promoter polymorphism with disease activity, and radiologic joint damage after 3, 6 and 9 years follow-up in a large (*n *= 208), well-characterized cohort of patients with early RA [26]. Next, we performed a meta-analysis to draw more definitive conclusions on the role of the *TNFA *-308G > A polymorphism in the severity of RA and radiological joint damage. This meta-analysis included a total of 2,053 patients and combined our data with previously published studies. To evaluate possible mechanisms underlying an association, we investigated gene expression in the different genotype groups in a cohort of 66 individuals with active RA.

## Material and methods

### Ethics statement

The *Commissie Mensgebonden Onderzoek (CMO) Regio Arnhem Nijmegen *of the Radboud University Nijmegen Medical Centre approved the study (CMO number 2004/014). All patients had provided written informed consent prior to participation in the study. All clinical investigations were conducted according to the principles expressed in the Declaration of Helsinki.

### Patients

All patients in this study fulfilled the 1987 American College of Rheumatology (ACR) criteria for RA and attended the Department of Rheumatology of the Radboud University Nijmegen Medical Centre or the St. Maartenshospital in Nijmegen, The Netherlands.

The impact of the *TNFA *-308G > A polymorphism on disease severity was analyzed using DNA isolated from blood of 208 patients from a long-term observational inception cohort with early RA [26]. Patients in this cohort have a disease duration < 1 year and no prior use of DMARDs at enrollment. Disease activity was assessed using the 28-joint disease activity score (DAS28) at each 3-monthly visit. Radiologic joint damage at baseline and after 1, 2, 3, 6 and 9 years of disease duration was measured using radiographs of the hands and feet, read in chronological order by one of four raters according to the Ratingen score. The latter is a validated modification of the Larsen score and evaluates joint surface destruction, graded from 0 to 5, in 38 separate hand and foot joints (range 0 - 190) [27]. The intra-class correlation coefficient (ICC) for inter-rater reliability was 0.85; this was tested previously with the four raters in 10 patients over 9 years.

*TNFA *expression analysis was assessed in RNA from blood of 66 additional patients with active RA as defined by DAS28 > 3.2. All patients attended the same hospital, and blood for RNA isolation was always collected between 9 and 10 o'clock in the morning to avoid potential circadian fluctuations.

### Molecular analysis

All DNA and RNA analyses were performed in a *Coördinatie Commissie ter bevordering van de Kwaliteitsbeheersing van het Laboratoriumonderzoek *(CCKL)-accredited laboratory at the department of Human Genetics at the Radboud University Nijmegen Medical Centre in Nijmegen.

### Genotyping of the -308G > A *TNFA *polymorphism

Genomic DNA was extracted from peripheral venous blood (10 ml/sample) according to standard protocols [28]. Both patient cohorts, thus, a total of 274 patients, were genotyped for the -308G > A (rs1800629) promoter polymorphism using a TaqMan genotyping assay (ID: C7514879_10) on the 7500 Fast Real-Time PCR system (Applied Biosystems, Foster City, CA, USA). Results were analyzed using Allelic Discrimination software version 1.4 (Applied Biosystems).

### RNA isolation, synthesis of cDNA and quantitative PCR

RNA was isolated from whole blood within 30 minutes after collection, using the RNeasy midi kit (Qiagen Benelux BV Venlo, The Netherlands). The quality and quantity of the purified RNA was checked on a NanoDrop spectrophotometer (Nanodrop technologies, Montchanin, DE, USA). Degradation of RNA was controlled for by agarose gel electrophoresis. Per sample, 1 μg of total RNA was reverse-transcribed using 200 ng random hexanucleotides (Invitrogen, Breda, The Netherlands) and 200 units of moloney murine leukemia virus (M-MLV) Reverse Transcriptase (Invitrogen). For quantitative expression analysis a predesigned and validated gene-specific probe-based TaqMan gene expression assay (ID: Hs00174128_m1) was used according to the manufacturer's protocol (Applied Biosystems). Samples were run on the 7500 Fast Real-Time PCR System (Applied Biosystems) using standard protocols. The general housekeeping gene *B2M *(Beta-2-microglobulin, TaqMan gene expression assay ID: Hs99999907_m1) was used as the endogenous control. Threshold cycle numbers (referred to as Ct) were obtained using the 7500 System SDS software version 1.4 (Applied Biosystems). All samples were measured twice, and duplicate samples with a SD larger than 0.5 were excluded from the analysis. The relative quantity (RQ) of the gene-specific mRNA was calculated from the average value of the ΔCt ((*TNFA *Ct) - (endogenous control gene Ct)). Differences in expression between two groups of samples were calculated by the 2^ΔΔCt ^method [29].

### Statistical analysis

Hardy-Weinberg equilibrium (HWE) was tested in both patient samples using the chi square test. Baseline differences between genotype groups were analyzed using the chi square test, Student's *t*-test or Wilcoxon test, as appropriate. Longitudinal analysis of radiologic joint damage in patients with RA was performed using generalized linear regression models with random coefficients (mixed models), allowing for analysis of repeated measures within a patient. The joint damage score was the dependent variable, genotype the independent variable, under addition of time, time squared and confounders. Regression assumptions were checked by evaluating the distribution of residuals and by a plot of observed and predicted joint damage scores. Mean differences in *TNFA *expression (ΔCt; (*TNFA *Ct) - (endogenous control gene Ct)) were analyzed using the independent Student's *t*-test.

A *P*-value < 0.05 was considered statistically significant in each situation. Analyses were performed using SPSS for Windows, version 14.0 (SPSS, Chicago, IL, USA) and SAS version 8.2 (SAS Institute Inc., Cary, NC, USA).

### Meta-analysis

Pubmed (up to March 31, 2012) was searched to identify publications investigating the effect of the -308G > A polymorphism on joint damage. Using the search terms: 1) RA AND TNF AND (polymorphism or -308); 2) RA AND joint damage AND (polymorphism or TNF or -308 and TNF; 3) RA and erosions in combination with polymorphism and TNF or -308. The literature search yielded 292 papers, 37 of which reported on relevant primary analysis of the *TNFA *-308G > A polymorphism. In order to be included in this meta-analysis, studies had to 1) include RA patients according to the ACR criteria, and 2) report on disease severity (presence or absence of erosions). Review manager 5 was used to perform a meta-analysis including 11 studies (10 published ones and this study) [1-10].

Homogeneity of odds ratios (ORs) among cohorts was calculated using the Breslow-Day method, and pooled ORs were calculated under a fixed and random effects model (Mantel-Haenszel meta-analysis).

## Results

### Relation of *TNFA *-308G > A with disease activity and radiologic joint damage

The frequency of the A allele in the inception cohort was 18.3%, genotype frequencies were 66.3% for the GG group, 30.8% for the GA group and 2.9% for the AA group. Due to the small AA genotype group, the GA and AA genotype groups were merged and analyzed as one group. Demographic and disease characteristics were similar in the GG and GA+AA genotype groups at baseline (Table 1). No significant differences in disease activity were observed between genotype groups at baseline (*P *= 0.38) and after 3 (*P *= 0.85), 6 (*P *= 0.1) and 9 (*P *= 0.12) years of follow-up.

**Table 1:** Demographics of the inception cohort (*n *= 208)

		GG	GA+AA	
	Number	(*n *= 138)	(*n *= 64 + 6)	*P*-value
Age, years, mean (SD)		52 (13)	50 (13)	0.21
Female gender, n (%)		89 (64%)	50 (71%)	0.32
RF positivity, n (%)		105 (76%)	55 (79%)	0.69
DAS28, mean (SD)				
Baseline	208	5.2 (1.4)	5.3 (1.3)	0.38
3-year average	208	4.0 (1.1)	3.9 (1.0)	0.85
6-year average	159	4.0 (1.0)	3.7 (1.0)	0.10
9-year average	115	4.0 (1.0)	3.7 (1.0)	0.12
Patients with erosions at baseline, n (%)	208	76 (55%)	44 (63%)	0.28
Ratingen score, median (range)				
Baseline	208	0 (0-2)	0 (0-2)	0.17
3 years	208	7 (0-15)	4 (0-10)	0.08
6 years	160	12 (2-26)	10 (1-21)	0.18
9 years	116	22 (10-38)	14 (3-24)	0.07

Percentages are expressed in relation to the total number of patients for each genotype group. RF, rheumatoid factor; DAS28, 28-joint disease activity score.

**Figure 1:**
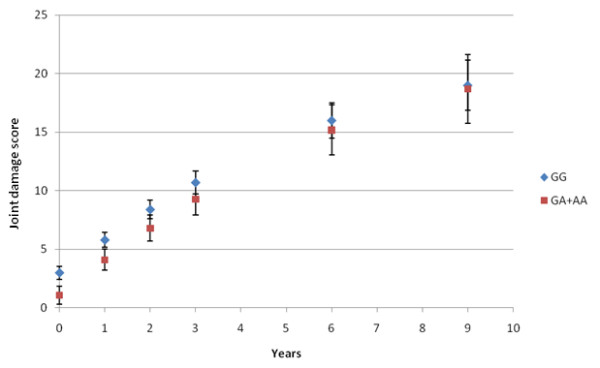
**Development of corrected joint scores over time showing the GG and GA+AA genotypes, separately**. Data points were calculated using the mixed model (least-square means and standard errors), including correction for age and 28-joint disease activity score (DAS28).

In the longitudinal regression analysis (mixed model) with radiologic joint damage as the dependent variable, a significant difference between the GG and GA+AA groups in the progression of joint damage was observed (Table 2 and Figure 1). As shown in Table 2, the progression of joint damage in the patients was 1.6 points per year, with progression rate slightly declining (-0.13 points) over the years of study. At baseline, the joint damage score of the genotype GA+AA group was on average 5 points lower (*P *= 0.002) than the score of the GG genotype group, corrected for age and DAS28 at baseline. There was no significant interaction between genotype and time of study, that is, the difference in joint damage score between genotype groups persisted during follow-up (*P *= 0.61), although in Figure 1 it appears that the joint damage scores of both groups converge at year-9.

**Table 2:** Longitudinal regression of joint damage and *TNFA *-308G > A genotype (*n *= 208)

Effect	Estimate	Standard error (estimate)	*P*-value
Intercept	5.8	1.18	< 0.0001
Genotype GA+AA	-5.0	1.62	**0.0020**
Year	1.6	0.38	< 0.0001
Year^2^	-0.13	0.020	< 0.0001
Genotype GA+AA × year	0.17	0.34	**0.61**

Shown are the variables in the longitudinal regression analysis over 9 years, with joint erosion score as the dependent variable and *TNFA *-308G > A or *TNFA *-308G > A × year as primary dependent variables. Year^2 ^allows for a non-linear course of joint progression score over time. The analysis was corrected for gender, and time-averaged 28-joint disease activity score (DAS28), rheumatoid factor and age were not confounders.

Since TNF might uncouple the relation between disease activity and joint damage [30], we assessed whether the use of TNF blocking agents was frequent in this patient population. The number of patients with past or present treatment with these agents was low, and was similar in both genotype groups (*n *= 20 (14%) and *n *= 12 (17%)) in the GG and GA+AA groups, respectively.

### Meta-analysis

**Figure 2:**
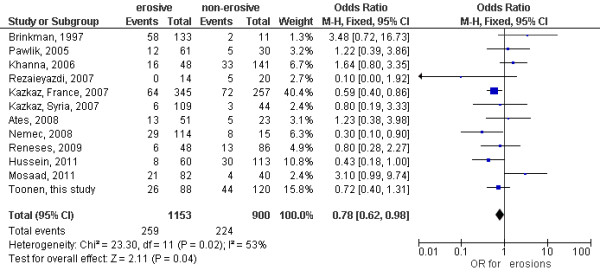
**Meta-analysis results for the TNF -308G > A variant**. Events indicate the number of patients with genotype GA or AA. M-H, Mantel-Haenszel.

A total of 2,053 RA patients from 10 published studies, as well as the current one, were included in a meta-analysis to further confirm the observed association of TNF -308G > A with joint damage, in this case defined as the presence or absence of erosions. Table 3 presents an overview of characteristics and the definition of erosions of the studies included in the meta-analysis. The analysis indeed confirmed that the -308G > A polymorphism is statistically significantly associated with the presence of erosions (*P *= 0.04, OR 0.78, 95% CI 0.62, 0.98) (Figure 2), with patients carrying the GA/AA genotypes having less erosions. As we observed moderate heterogeneity, we also applied a random effects meta-analysis, producing a similar OR (0.86, 95% CI 0.58, 1.29).

**Table 3:** Overview of the published studies included in the meta-analysis

Reference	Population	N	Female, %	Age, years	**Disease duration**, years	Scoring method	Definition erosive disease
Brinkman [2]	Dutch	144	65	66.8	14.06	ns	Erosions on any radiograph during first 3 years
Pawlik [10]	Polish	91	63	51.7 (range 21-74)^#^	4.7 ± 2.1	ns	Erosions in chest, hands or feet
Khanna [9]	Americans (Western US) and Mexican	189	76	ns	0.47 (0.3-0.71)*	Sharp	Erosion score ≥ 2 In hand/wrist or feet
Rezaieyazdi [7]	Iranian	34	n.s.	47.3 ± 13.8	4.0 ± 3.7	ns	Erosions in hands
Kazkaz [8]	Syrian	156	80	44.02 ± 14.47	7.28 ± 4.87	Larsen	Destruction score ≥ 2, right wrist
Kazkaz [8]	French	512	75	57.56 ± 14.67	10.05 ± 8.66	Larsen	Destruction score ≥ 2, right wrist
Ates [5]	Turkish	98	80	50.8 ± 12	ns	ns	Erosions in hands or feet
Nemec [6]	Caucasians from Moravian regions of the Czech Republic	130	75	56.21 (20.25-79)*	11.08 (2.08-50.17)*	Steinbrocker	Erosions of stage II-IV in hands
Reneses [4]	Spanish	134	70	50.4 ± 1.3	< 1	Sharp van der Heijde	Erosion at 1 year in hands, wrist or feet
Hussein [3]	Egyptian	172	100	47.4 ± 9.3	10.65 ± 7.9	Sharp	≥ 1 erosions in peripheral joints (hands or feet)
Mosaad [1]	Egyptian	122	80	39.9 ± 10.3	5.9 ± 5.1	Steinbrocker	Erosions in hands

Age and disease duration are depicted as mean ± SD unless stated otherwise. *Median (range); ^#^mean (range); ns, not specified.

### *TNFA *expression and the role of the -308G > A promoter polymorphism

**Figure 3:**
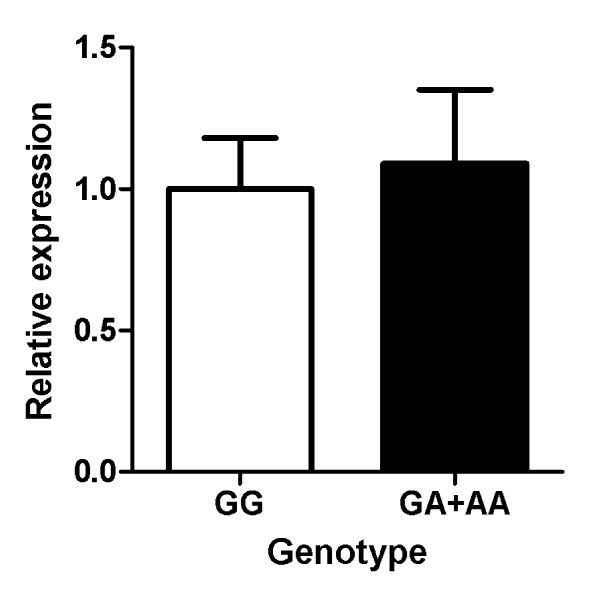
**Relative expression of the *TNFA *gene per genotype group**. The GA genotype group (*n *= 15) and the AA genotype group (*n *= 2) were combined and compared to the GG genotype group (*n *= 49). No difference in *TNFA *expression was observed between the GG group and the GA+AA group (*p *= 0.420).

To assess whether the difference in radiologic joint damage between G-homozygotes and A-allele carriers could be explained at the level of expression of this cytokine, we measured the RNA expression levels of *TNFA *dependent on -308G > A genotype in a sample of 66 patients with active RA. Among the sixty-six patients, forty-nine carried the GG genotype, fifteen were GA-heterozygotes, and two were A-homozygotes. Again, the GA and AA genotypes were merged and analyzed as one group. No difference in *TNFA *expression was observed between the GG group when compared to the GA+AA group (*P *= 0.420) (Figure 3).

## Discussion

Based on earlier literature, we tested the hypothesis that in RA, disease severity defined by the average DAS28 and radiologic joint damage, may be influenced by the *TNFA *-308G > A genotype. Our own study and the meta-analysis indeed confirm that patients carrying the common GG genotype have worse radiologic outcomes. This could not be explained by differences in either disease activity, genotype-dependent *TNFA *expression, or the use of TNF blocking agents.

Our results show that the GG genotype is associated with a worse radiologic outcome, in spite of the fact that in our early inception cohort the radiologic progression after 9 years was similar in both genotypes. It would be of great interest to see if these results could be replicated in other early RA cohorts though the number of published studies hitherto is too small for a meta-analysis.

Many studies investigating the relationship between the -308G > A promoter polymorphism and severity of RA and radiological joint damage report contradictive results [1-14]. Therefore, a meta-analysis offers a good alternative for evaluating the presence or absence of an association in situations where individual studies are inconclusive. Our meta-analysis indeed confirmed that the -308G > A polymorphism is associated with the presence of erosions, with patients carrying the GA/AA genotypes having fewer erosions. Although the result of the meta-analysis is consistent with the results of our primary study, it is important to mention that there was significant heterogeneity in the studies included in the meta-analysis (*I*^2 ^= 53%). This could be caused by differences in study design, mean duration of RA, sample size and ethnicity. An additional reason is the assessment of the presence of erosions, which was assessed in radiographs of different joints (see Table 3), though most studies included erosions in the hands. Also the definition of erosive disease differed between the studies, but the outcome used in the current study was the presence of any erosion in the joints examined. As we observed heterogeneity we also performed a random-effects meta-analysis. This yielded a similar OR but, as expected, a larger CI. Even so, both analyses point to a modest effect of the polymorphism on the development of joint damage.

In this study we also assessed whether the difference in radiologic joint damage between G- homozygotes and A-allele carriers could be explained by *TNFA *allele-specific differences in mRNA expression. Many studies have focused on the potential allele-specific expression of the *TNFA *gene and TNFα production. Helmig and co-workers reported that the -308G allele is associated with *TNFA *expression in a cohort of 178 healthy subjects [23]. In contrast, a recent meta-analysis in which 1,825 healthy subjects were investigated, reported no association between the -308G > A polymorphism and TNF protein and mRNA levels [25]. However, this might still be the case for persons suffering from a disease. For instance, the study of Muoz-Valle, *et al*. (50 patients with osteoarthritis), and the study of Oregon-Romero, *et al*. (50 patients with RA) both report an association between the -308G allele and higher *TNFA *expression levels compared to the -308A allele [21,22]. Nonetheless, our study does not support this hypothesis and shows that the effect of the -308G > A variant on joint damage is not explained by allele-dependent expression in RA. Linkage disequilibrium (LD) could be an overall factor to explain this incongruent result if we assume that the -308G > A variant is not functional. In fact, LD is strong in this area of the genome, and it is difficult to study the role of a single nucleotide polymorphism (SNP) in this region. Nevertheless, a published haplotype analysis including three SNPs (-857C > T, -308G > A, and -238G > A) in the promoter of the *TNFA *gene also showed no association between the SNPs and circulating TNF levels or *TNFA *mRNA expression [25]. It is known that the LD in the *TNFA *region even extends much further and is in strong LD with the shared epitope (SE) [31]. This could explain some of the inconsistency in the findings, although the results of a study of Khanna and co-workers indicates an effect of the -308G > A polymorphism on joint damage progression independently of the SE [9]. Our patients are not genotyped for the SE and we could not stratify for SE.

## Conclusions

Our data support an association between the *TNFA *-308GG genotype and joint damage, but not with disease activity or *TNFA *expression. Our meta-analysis shows that the effects of this polymorphism are modest, which makes it unsuitable as a single predictive marker for disease severity.

## Abbreviations

ACR: American College of Rheumatology; CCKL: *Coördinatie Commissie ter bevordering van de Kwaliteitsbeheersing van het Laboratoriumonderzoek*; cDNA: complementary DNA; Ct: Cycle threshold; DAS28: 28-joint disease activity score; DMARD: disease-modifying antirheumatic drug; HWE: Hardy-Weinberg equilibrium; ICC: intra-class correlation coefficient; LD: linkage disequilibrium; M-MLV: moloney murine leukemia virus; OR: odds ratio; PCR: polymerase chain reaction; RA: rheumatoid arthritis; RQ: relative quantity; SE: shared epitope; SNP: single nucleotide polymorphism; TNF: tumor necrosis factor.

## Competing interests

The authors declare that they have no competing interests.

## Authors' contributions

ET collected DNA and RNA samples from RA patients, carried out and interpreted the results of the molecular genetic studies, participated in the study design and wrote the manuscript. PB participated in the study design and evaluated the manuscript. JF carried out the longitudinal regression analysis and evaluated the manuscript. AdB helped perform the molecular genetic studies, interpreted the results and evaluated the manuscript. AE was involved in collecting DNA and RNA samples from RA patients and evaluated the manuscript. PM helped in collecting data for the meta-analysis and evaluated the manuscript. HM helped in collecting data for the meta-analysis and evaluated the manuscript. HS interpreted the results and evaluated the manuscript. PvR participated in the study design, interpreted the results and evaluated the manuscript. BF participated in the study design, was involved in interpreting the results and evaluated the manuscript. MC was involved in collecting DNA and RNA samples from RA patients, participated in the study design, collected data for the meta-analysis, interpreted the results and wrote the manuscript. All authors read and approved the final manuscript.

## References

[B1] Mosaad YM Abdelsalam A El-Bassiony SR Association of tumour necrosis factor-alpha -308 G/A promoter polymorphism with susceptibility and disease profile of rheumatoid arthritis Int J Immunogenet 2011 14 427 433 10.1111/j.1744-313X.2011.01028.x 21806780

[B2] Brinkman BM Huizinga TW Kurban SS van der Velde Schreuder GM Hazes JM Breedveld FC Verweij CL Tumour necrosis factor alpha gene polymorphisms in rheumatoid arthritis: association with susceptibility to, or severity of, disease? Br J Rheumatol 1997 14 516 521 10.1093/rheumatology/36.5.516 9189051

[B3] Hussein YM Mohamed RH Pasha HF El-Shahawy EE Alzahrani SS Association of tumor necrosis factor alpha and its receptor polymorphisms with rheumatoid arthritis in female patients Cell Immunol 2011 14 192 196 10.1016/j.cellimm.2011.06.023 21777909

[B4] Reneses S Gonzalez-Escribano MF Fernandez-Suarez A Pestana L Davila B Wichmann I Garcia A The value of HLA-DRB1 shared epitope, -308 tumor necrosis factor-alpha gene promoter polymorphism, rheumatoid factor, anti-citrullinated peptide antibodies, and early erosions for predicting radiological outcome in recent-onset rheumatoid arthritis J Rheumatol 2009 14 1143 1149 10.3899/jrheum.081075 19411391

[B5] Ates O Hatemi G Hamuryudan V Topal-Sarikaya A Tumor necrosis factor-alpha and interleukin-10 gene promoter polymorphisms in Turkish rheumatoid arthritis patients Clin Rheumatol 2008 14 1243 1248 10.1007/s10067-008-0893-1 18427872

[B6] Nemec P Pavkova-Goldbergova M Stouracova M Vasku A Soucek M Gatterova J Polymorphism in the tumor necrosis factor-alpha gene promoter is associated with severity of rheumatoid arthritis in the Czech population Clin Rheumatol 2008 14 59 65 10.1007/s10067-008-0924-y 17562093

[B7] Rezaieyazdi Z Afshari JT Sandooghi M Mohajer F Tumour necrosis factor a -308 promoter polymorphism in patients with rheumatoid arthritis Rheumatol Int 2007 14 189 191 10.1007/s00296-007-0444-0 17763852

[B8] Kazkaz L Marotte H Hamwi M Angelique CM Roy P Mougin B Miossec P Rheumatoid arthritis and genetic markers in Syrian and French populations: different effect of the shared epitope Ann Rheum Dis 2007 14 195 201 17068065 10.1136/ard.2004.033829 PMC1798494

[B9] Khanna D Wu H Park G Gersuk V Gold RH Nepom GT Wong WK Sharp JT Reed EF Paulus HE Tsao BP Association of tumor necrosis factor alpha polymorphism, but not the shared epitope, with increased radiographic progression in a seropositive rheumatoid arthritis inception cohort Arthritis Rheum 2006 14 1105 1116 10.1002/art.21750 16572445

[B10] Pawlik A Florczak M Ostanek L Brzosko M Brzosko I Szklarz BG TNF-alpha -308 promoter polymorphism in patients with rheumatoid arthritis Scand J Rheumatol 2005 14 22 26 15903021

[B11] Rojas-Villarraga A Diaz FJ Calvo-Paramo E Salazar JC Iglesias-Gamarra A Mantilla RD Anaya JM Familial disease, the HLA-DRB1 shared epitope and anti-CCP antibodies influence time at appearance of substantial joint damage in rheumatoid arthritis J Autoimmun 2009 14 64 69 10.1016/j.jaut.2008.11.004 19117726

[B12] Lacki JK Moser R Korczowska I Mackiewicz S Muller W TNF-alpha gene polymorphism does not affect the clinical and radiological outcome of rheumatoid arthritis Rheumatol Int 2000 14 137 140 10.1007/s002960050117 10836523

[B13] Fonseca JE Cavaleiro J Teles J Sousa E Andreozzi VL Antunes M maral-Turkman MA Canhao H Mourao AF Lopes J Caetano-Lopes J Weinmann P Sobral M Nero P Saavedra MJ Malcata A Cruz M Melo R Braña A Miranda L Patto JV Barcelos A da Silva JC Santos LM Figueiredo G Rodrigues M Jesus H Quintal A Carvalho T da Silva JA Contribution for new genetic markers of rheumatoid arthritis activity and severity: sequencing of the tumor necrosis factor-alpha gene promoter Arthritis Res Ther 2007 14 R37 10.1186/ar2173 17408492 PMC1906815

[B14] Wilson AG de VN van de Putte LB Duff GW A tumour necrosis factor alpha polymorphism is not associated with rheumatoid arthritis Ann Rheum Dis 1995 14 601 603 10.1136/ard.54.7.601 7668906 PMC1009943

[B15] Bouma G Crusius JB Oudkerk PM Kolkman JJ von Blomberg BM Kostense PJ Giphart MJ Schreuder GM Meuwissen SG Pena AS Secretion of tumour necrosis factor alpha and lymphotoxin alpha in relation to polymorphisms in the TNF genes and HLA-DR alleles. Relevance for inflammatory bowel disease Scand J Immunol 1996 14 456 463 10.1046/j.1365-3083.1996.d01-65.x 8668926

[B16] Hajeer AH Hutchinson IV Influence of TNFalpha gene polymorphisms on TNFalpha production and disease Hum Immunol 2001 14 1191 1199 10.1016/S0198-8859(01)00322-6 11704281

[B17] Abraham LJ Kroeger KM Impact of the -308 TNF promoter polymorphism on the transcriptional regulation of the TNF gene: relevance to disease J Leukoc Biol 1999 14 562 566 10534109 10.1002/jlb.66.4.562

[B18] Kroeger KM Abraham LJ Identification of an AP-2 element in the -323 to -285 region of the TNF-alpha gene Biochem Mol Biol Int 1996 14 43 51 8886268 10.1080/15216549600201512

[B19] Kroeger KM Carville KS Abraham LJ The -308 tumor necrosis factor-alpha promoter polymorphism effects transcription Mol Immunol 1997 14 391 399 10.1016/S0161-5890(97)00052-7 9293772

[B20] Braun N Michel U Ernst BP Metzner R Bitsch A Weber F Rieckmann P Gene polymorphism at position -308 of the tumor-necrosis-factor-alpha (TNF-alpha) in multiple sclerosis and it's influence on the regulation of TNF-alpha production Neurosci Lett 1996 14 75 78 8887999

[B21] Oregon-Romero E Vazquez-Del MM Ruiz-Quezada SL Navarro-Hernandez RE Rangel-Villalobos H Martinez-Bonilla G Bernard-Medina AG rmendariz-Borunda J Garcia-Banuelos J Munoz-Valle JF Tumor necrosis factor alpha-308 and -238 polymorphisms in rheumatoid arthritis. Association with messenger RNA expression and sTNF-alpha J Investig Med 2008 14 937 943 18797411 10.2310/JIM.0b013e318189152b

[B22] Munoz-Valle JF Oregon-Romero E Rangel-Villalobos H Martinez-Bonilla GE Castaneda-Saucedo E Salgado-Goytia L Leyva-Vazquez MA Illades-Aguiar B arcon-Romero LD Espinoza-Rojo M Parra-Rojas I High expression of TNF alpha is associated with -308 and -238 TNF alpha polymorphisms in knee osteoarthritis Clin Exp Med 2012 10.1007/s10238-012-0216-3 23108479

[B23] Helmig S Aliahmadi N Stephan P Dohrel J Schneider J TNF-alpha -308 genotypes are associated with TNF-alpha and TGF-beta(1) mRNA expression in blood leucocytes of humans Cytokine 2011 14 306 310 10.1016/j.cyto.2010.11.018 21169032

[B24] Knight JC Keating BJ Rockett KA Kwiatkowski DP In vivo characterization of regulatory polymorphisms by allele-specific quantification of RNA polymerase loading Nat Genet 2003 14 469 475 10.1038/ng1124 12627232

[B25] Mekinian A Tamouza R Pavy S Gestermann N Ittah M Mariette X Miceli-Richard C Functional study of TNF-alpha promoter polymorphisms: literature review and meta-analysis Eur Cytokine Netw 2011 14 88 102 21768061 10.1684/ecn.2011.0285

[B26] Welsing PM van Riel PL The Nijmegen inception cohort of early rheumatoid arthritis J Rheumatol Suppl 2004 14 14 21 15053447

[B27] Rau R Wassenberg S Herborn G Stucki G Gebler A A new method of scoring radiographic change in rheumatoid arthritis J Rheumatol 1998 14 2094 2107 9818650

[B28] Miller SA Dykes DD Polesky HF A simple salting out procedure for extracting DNA from human nucleated cells Nucleic Acids Res 1988 14 1215 10.1093/nar/16.3.1215 3344216 PMC334765

[B29] Livak KJ Schmittgen TD Analysis of relative gene expression data using real-time quantitative PCR and the 2(-Delta Delta C(T)) Method Methods 2001 14 402 408 10.1006/meth.2001.1262 11846609

[B30] van den Berg WB van Riel PL Uncoupling of inflammation and destruction in rheumatoid arthritis: myth or reality? Arthritis Rheum 2005 14 995 999 10.1002/art.20981 15818666

[B31] Jawaheer D Li W Graham RR Chen W Damle A Xiao X Monteiro J Khalili H Lee A Lundsten R Begovich A Bugawan T Erlich H Elder JT Criswell LA Seldin MF Amos CI Behrens TW Gregersen PK Dissecting the genetic complexity of the association between human leukocyte antigens and rheumatoid arthritis Am J Hum Genet 2002 14 585 594 10.1086/342407 12181776 PMC449696

